# Prolonged enhancement of cytotoxic T lymphocytes in the post-recovery state of severe COVID-19

**DOI:** 10.1186/s40560-021-00591-3

**Published:** 2021-12-20

**Authors:** Yumi Mitsuyama, Kazuma Yamakawa, Katsuhide Kayano, Miho Maruyama, Takeshi Wada, Satoshi Fujimi

**Affiliations:** 1 Division of Trauma and Surgical Critical Care, Osaka General Medical Center, Osaka, Japan; 2 Department of Emergency Medicine, Osaka Medical and Pharmaceutical University, 2-7 Daigakumachi, Takatsuki, Osaka 569-8686 Japan; 3 Division of Acute and Critical Care Medicine, Department of Anesthesiology and Critical Care Medicine, Faculty of Medicine, Hokkaido University, Sapporo, Japan

**Keywords:** COVID-19, Post-recovery, Single-cell mass cytometry, T-bet, Granzyme B, Cytotoxic T lymphocytes

## Abstract

We evaluated the peripheral blood immune responses of lymphocytes in severe Coronavirus disease 2019 (COVID-19) patients in different stages of recovery using single-cell mass cytometry. The patients with prolonged hospitalization did not show recovery of B lymphocyte counts and CD4-positive T lymphocyte counts but did show abundant CD8-positive T lymphocytes. CD4 and CD8 T cells expressing high levels of T-bet and Granzyme B were more abundant in post-recovery patients. This study showed that cytotoxic Th1 and CD8 T cells are recruited to the peripheral blood long after recovery from COVID-19.

## Dear Editor,

The pandemic of coronavirus disease 2019 (COVID-19) is a global public health emergency. Several studies have reported a complex network of peripheral blood immune responses in patients with COVID-19 [1]. However, very little is known about immune cell alterations in critically ill patients who have recovered from COVID-19.

We profiled the characteristic peripheral cellular profiles of patients with COVID-19 using single-cell mass cytometry (cytometry by time-of-flight: CyTOF). Peripheral blood mononuclear cells (PBMCs) of six patients who recovered from severe COVID-19, three of whom were discharged (recovered patients: RP) and three who required prolonged hospitalization (hospitalized patients: HP) at the time of blood sampling, were compared with those of healthy donors (HD) (Table 1). Patients’ blood samples were collected about 3 months after admission.

**Table 1:** Patient characteristics

	Hospitalized patients^b^			Recovered patients			Healthy donors			
	Patient 1	Patient 2	Patient 3	Patient 4	Patient 5	Patient 6	Donor 1	Donor 2	Donor 3	Donor 4
Age, years	75	85	73	67	57	62	65	62	71	70
Sex	Female	Male	Male	Male	Male	Female	Female	Female	Male	Male
Body mass index, kg/m^2^	20.5	22.7	20.9	24.5	23	29.1	18.7	18.6	21.4	26.9
Past medical history	None	HT	None	DM	None	None				
Clinical features at admission										
Severity of ARDS^a^	Moderate	Severe	Severe	Moderate	Severe	Severe				
APACHE II score	18	17	20	10	9	16				
Disease course										
ECMO	−	−	−	−	+	+				
Tracheostomy	+	+	+	+	−	−				
Length of stay in ICU, days	37	29	45	27	17	18				
Days of mechanical ventilation, days	42	37	45	34	16	17				
Length of stay in hospital, days	156	141	187	92	31	67				
Discharge	Nursing home	Home	Home	Home	Home	Home				
Days from hospitalization to specimen	100	103	98	105	95	87				

*ARDS* acute diffuse respiratory syndrome, *APACHE* acute physiology and chronic health evaluation, *ECMO* extracorporeal membranous oxygenation, *ICU* intensive care unit, *DM* diabetes mellitus, *HT* hypertension

^a^Severe: 100 ≤ PaO_2_/FiO_2_, moderate: 100 < PaO_2_/FiO_2_ ≤ 200 (on positive end-expiratory pressure of 5 cmH_2_O)

^b^Patients who were hospitalized at the time of blood sample collection

**Fig. 1:**
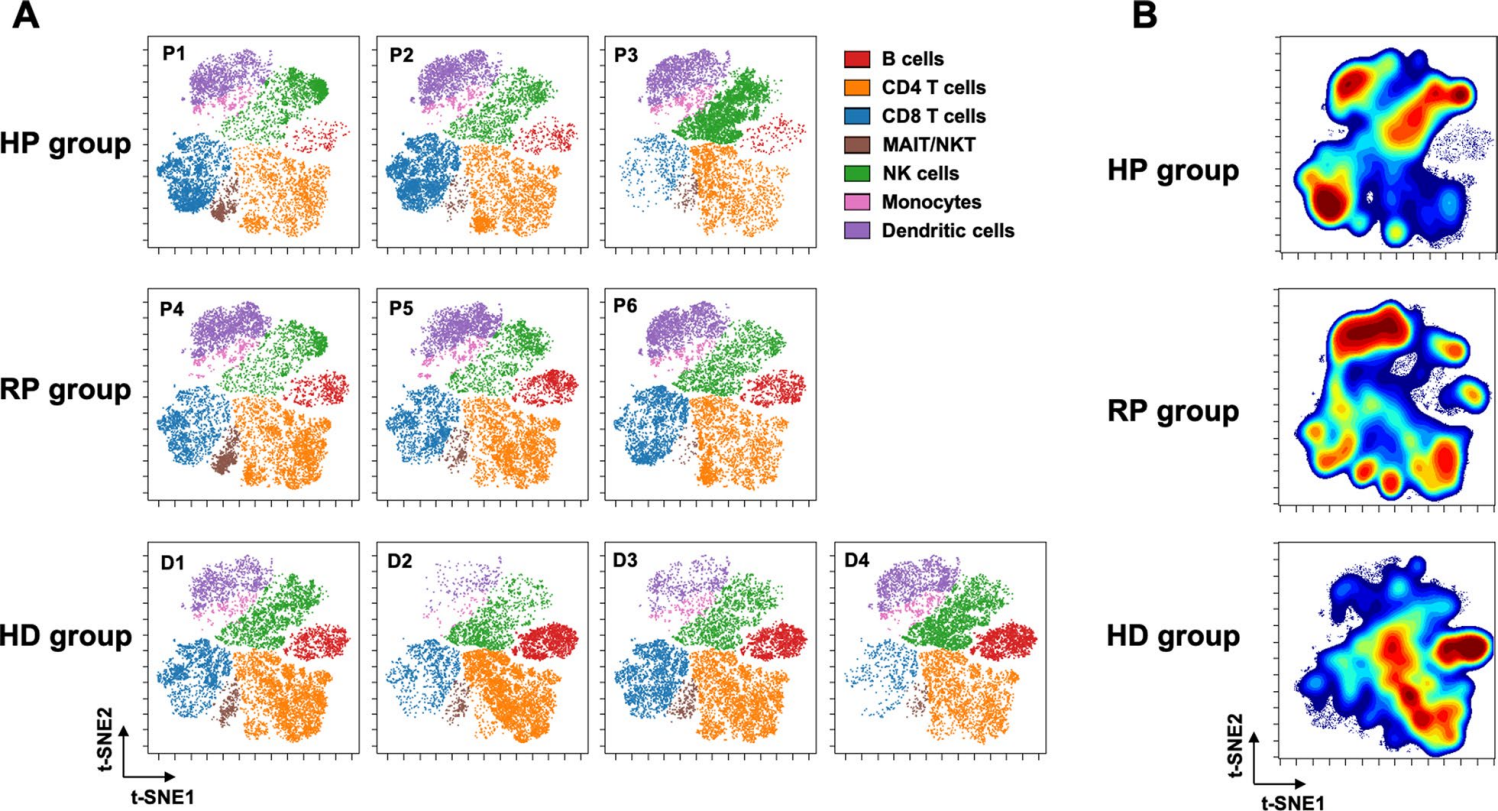
Characterization of peripheral blood mononuclear cells (PBMCs) in patients with COVID-19 and healthy donors. **A** The PBMC samples of all patients and the healthy donors were merged, and cell populations defined by the manual gating strategy were projected onto a t-SNE map and assigned specific colors. **B** Patient data within each group were concatenated by group, and CyTOF staining data for the three groups from CD45 + cells were analyzed by t-SNE and plotted as density contour plots. *B cells* B lymphocytes, *CD4 T cells* CD4 T lymphocytes, *CD8 T cells* CD8 T lymphocytes, *D* donor, *HD* healthy donors, *HP* hospitalized patients, *MAIT* mucosal-associated invariant T cells, *NK* natural killer cells, *NKT* natural killer T cells, *HP* hospitalized patient, *RP* recovered patient, *HD* healthy donor,  *t-SNE* t-distributed Stochastic Neighbor Embedding

**Fig. 2:**
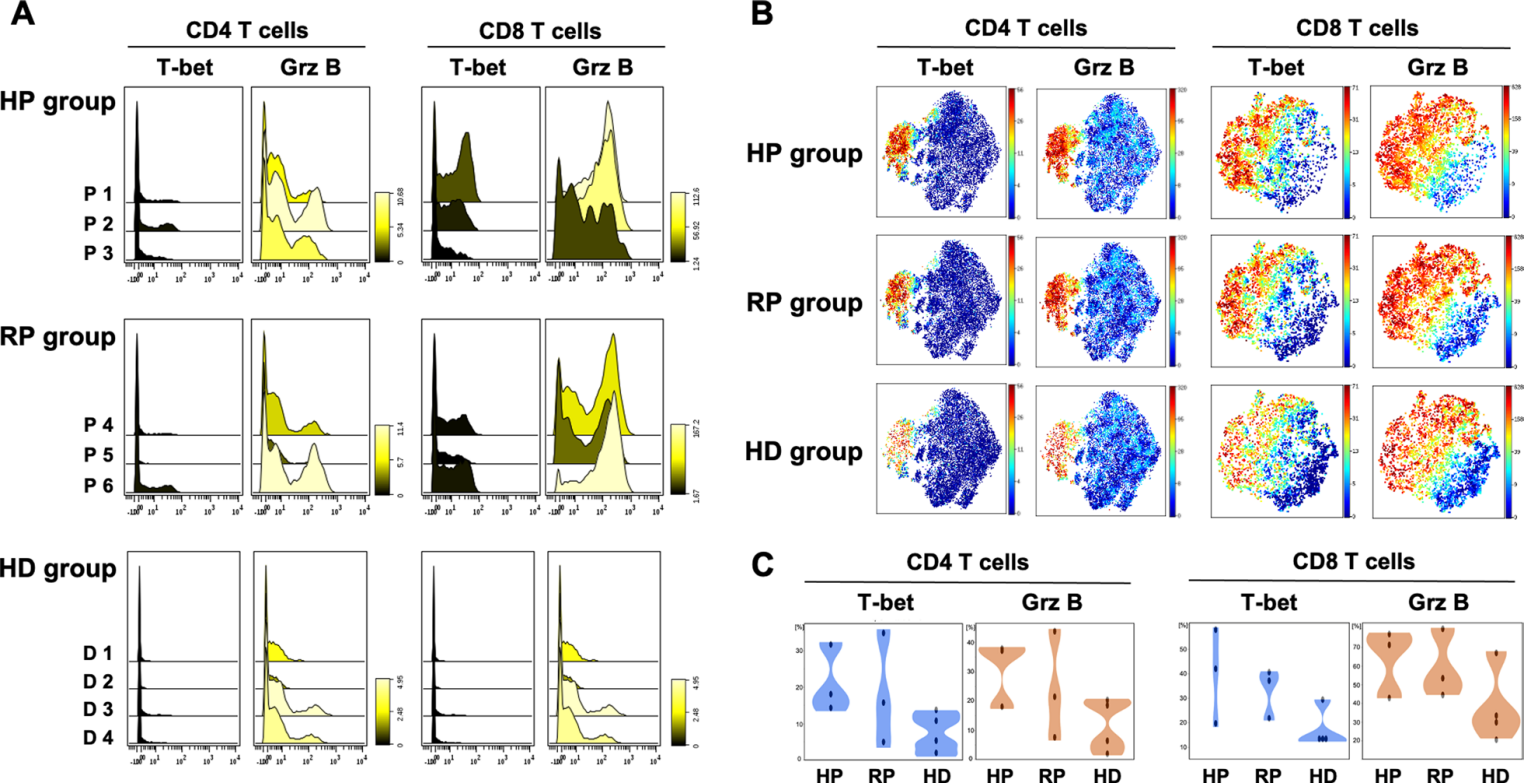
Expression of T-bet and Granzyme B on CD4 and CD8 T cells in patients with COVID-19 and healthy donors. **A** Histograms showing relative changes in expression intensities of the indicated markers on CD4 and CD8 T cells from the PBMCs of the patients and healthy donors. The color bar legend indicates differences in marker expression levels: yellow (increased) or black (decreased). **B** t-SNE projections of CD4 and CD8 T cells from the HP, RP and HD groups generated by concatenating patient data within each group for the indicated cell markers. **C** Violin plots showing the percentage of cells expressing the indicated markers in the three groups. The plots show the distribution of sample values. *CD4 T cells* CD4 T lymphocytes, *CD8 T cells* CD8 T lymphocytes, *Grz B* granzyme B, *HD* healthy donor, *HP* hospitalized patient, *PMBCs* peripheral blood mononuclear cells, *RP* recovered patient, *tSNE* t-distributed Stochastic Neighbor Embedding

A 43-marker antibody panel was used for CyTOF staining of PBMCs, which were analyzed on a Helios mass cytometer (Fluidigm Sciences Inc.). We identified seven cell subsets and visualized the changes in the cell populations of all samples on a t-distributed Stochastic Neighbor Embedding (t-SNE) map (Fig. 1A). For comparison of the three groups, data were concatenated within a group and the cell distribution was visualized on a t-SNE map (Fig. 1B). The contour density of B lymphocytes was lower in the HP group than that in the HD group, whereas that in the RP group had recovered to the same level as that in the HD group. CD4-positive T lymphocytes were fewer in the HP group, followed by the RP group, whereas CD8-positive T lymphocytes were more abundant in the RP and HP groups versus HD group. Natural killer (NK) cells were more abundant in the HP group, followed by the HD group, and were less frequent in the RP group. The frequency of protein expression inside and outside the cells of all samples is shown in the histogram (Fig. 2A). The data for each group were concatenated (Fig. 2B). CD4 T cells showing high expression of T-bet and Granzyme B were more abundant in the HP group, followed by the RP group. The number of CD8 T cells highly expressing T-bet and Granzyme B was also higher in the HP and RP groups (Fig. 2C).

We have shown the peripheral blood immune responses of lymphocytes and NK cells in severe COVID-19 patients in different stages of recovery. It has been reported that especially regarding B lymphocyte counts, lymphopenia of patients with acute-phase COVID-19 recovers after polymerase chain reaction tests become negative [2]. Our results suggest that long-term recovery of B lymphocytes might be related to the severity of illness and the current stage of recovery. The NK cell population is reported to be greatly altered in patients with acute COVID-19, with an expansion of the cytokine-producing NK cells and a decrease in the cytolytic NK cells responsible for innate immunity [3]. Although cytolytic NK cells recovered with improvement of the disease, the frequency of cytokine-producing NK cells remained elevated in severe COVID-19. Elevated cytokine-producing NK cells may lead to impaired NK cell cytotoxicity and decreased regulation of cellular and humoral adaptive immune responses [4].

T-bet is the master transcription factor of CD4 T helper type 1 (Th1) cells and plays a major part in protective immunity in cooperation with CD4, CD8 T cells and natural killer T (NKT) cells [5]. Granzyme B is mainly expressed on activated memory CD8 and memory CD4 T cells, NK cells and NKT cells during infection and inflammation, and has important roles in promoting removal of virus-infected cells by cytotoxic T cells and in suppressing the host immune response [6]. Therefore, the high expression of T-bet and Granzyme B in CD4 and CD8 T cells indicates increased cytotoxicity of lymphocytes. Previous reports showed that cytotoxicity is enhanced in CD4 and CD8 T cells in the acute phase of severe COVID-19, and the elevation in cytotoxic CD8 T cell counts persists after recovery [7, 8]. The present study also revealed that the persistence of T cells highly expressing T-bet and Granzyme B in the recovered COVID-19 patients might indicate prolonged suppression of the immune response and an unrecovered inflammatory process. Comparing the RP and HP groups, although recovery of B lymphocytes was restored in the RP group, the recruitment of cytotoxic T cells to the peripheral blood persisted in both post-COVID-19 groups. This may suggest that the activation of cellular immunity is more prolonged than that of humoral immunity in COVID-19, depending on the severity of the illness and the stage of recovery. These results suggest that restoration of immune homeostasis after COVID-19 may require a long time and that complications, such as secondary infections during the recovery process need to be addressed. Thus, the prolonged cytotoxicity of lymphocytes after recovery from COVID-19 may have implications for elucidation of the long-term changes in immune responses after COVID-19.

## Data Availability

The datasets analyzed during the current study are available from the corresponding author on reasonable request.

## Abbreviations

COVID-19: Coronavirus disease 2019
CyTOF: Cytometry by time-of-flight
NK cells: Natural killer cells
NKT cells: Natural killer T cells
PBMCs: Peripheral blood mononuclear cells
t-SNE: t-distributed Stochastic Neighbor Embedding
Th1: T helper type 1
